# Insulin augments tumor necrosis factor-alpha stimulated expression of vascular cell adhesion molecule-1 in vascular endothelial cells

**DOI:** 10.1186/1476-9255-8-34

**Published:** 2011-11-17

**Authors:** Daniel Z Mackesy, Marc L Goalstone

**Affiliations:** 1 Department of Research Service, Eastern Colorado Health Care System, 1055 Clermont Street, Denver, 80220, USA; 2 Department of Medicine, University of Colorado Denver, 12631 E. 17th Ave., Aurora, 80045, USA

**Keywords:** Tumor necrosis factor-alpha, inflammation, Vascular Adhesion Molecule-1, Nuclear Factor kappa-B, hyperinsulinemia, atherosclerosis

## Abstract

**Background:**

Atherosclerosis is an inflammatory disease that is marked by increased presence of Tumor Necrosis Factor-alpha (TNFα), increased expression of Vascular Cell Adhesion Molecule-1 (VCAM-1), increased presence of serum monocytes and activation of the canonical inflammatory molecule, Nuclear Factor Kappa-B (NFκB). Hyperinsulinemia is a hallmark of insulin resistance and may play a key role in this inflammatory process.

**Methods:**

Using Western blot analysis, immunocytochemistry, flow cytometry and biochemical inhibitors, we measured changes in VCAM-1 protein expression and NFκB translocation in vascular endothelial cells in the presence of TNFα and/or hyperinsulinemia and in the absence or presence of kinase pathway inhibitors.

**Results:**

We report that hyperinsulinemia augmented TNFα stimulated increases in VCAM-1 protein greater than seen with TNFα alone and decreased the time in which VCAM-1 translocated to the cell surface. We also observed that in the presence of Wortmannin, a biochemical inhibitor of phosphatidylinositol 3-kinase (a hallmark of insulin resistance), VCAM-1 expression was greater in the presence of TNFα plus insulin as compared to that seen with insulin or TNFα alone. Additionally, nuclear import of NFκB occurred sooner in the presence of insulin and TNFα together as compared to each alone, and in the presence of Wortmannin, nuclear import of NFκB was greater than that seen with insulin and TNFα alone.

**Conclusions:**

hyperinsulinemia and insulin resistance appear to augment the inflammatory effects of TNFα on VCAM-1 expression and NFκB translocation, both of which are markers of inflammation in the vasculature.

## Introduction

Type-2 Diabetes Mellitus (T2DM) is a constellation of disorders that includes, but is not limited to, hyperinsulinemia, dyslipidemia and insulin resistance. These pathologies are risk factors for retinopathy, neuropathy and cardio-vascular events, to name a few [1]. Vascular complications are the leading cause of morbidity and mortality in patients with diabetes.

Atherosclerosis is a major consequence of vascular dysfunction and in part comes from a collection of players that leads to, vascular smooth cell proliferation, lack of vascular compliance, endothelial cell remodeling, and increased response to inflammatory cytokines. One particular characteristic of atherogenesis is the increased expression of cellular adhesion molecules (CAMs) at the surface of vascular endothelial cells [2-4].

Although insulin is considered to be an anti-atherogenic hormone [5], other studies have suggested that long-term (i.e., chronic) insulin resistance accompanied by hyperinsulinemia contributes to the pathogenesis of atherosclerosis by augmenting the effects of inflammatory cytokines, thereby significantly increasing the expression of CAMs [6-11].

One such cytokine is tumor necrosis factor-alpha (TNFα). TNFα is secreted by mature macrophages and endothelial cells during the progression of atherosclerosis. Interestingly, TNFα activity is linked to insulin resistance [12], and many of these events are mediated in part by the pathways associated with extracellular signal-regulated kinases (ERK), c-jun N-terminal kinases (JNK) and nuclear factor kappa-B (NFκB) [13].

Among a myriad of effects, TNFα stimulates the increased expression of the cellular adhesion molecule, vascular cell adhesion molecule-1 (VCAM-1) [14]. In response to TNFα, upregulation of VCAM-1 increases the likelihood that serum-associated monocytes will adhere to the arterial endothelium, transmigrate from the intima to the media, and secrete both TNFα and other inflammatory cytokines; essentially promoting a positive feed-back process.

The question remains, however, does insulin in the context of insulin resistance/hyperinsulinemia exacerbate or mitigate the existing conditions of TNFα-stimulated VCAM-1 expression? Moreover, what are the molecular mechanism(s) that play a role in this process? Insulin resistance is frequently defined in molecular terminology as a post-insulin receptor dysfunction. It is commonly believed that perturbation of the phosphatidylinositol-3 kinase (PI3K) and Akt signal pathway leads to dysfunction in intracellular insulin signaling: a down regulation of translocation of glucose transporters to the membrane and decreased uptake of glucose. Yet, there may be other effects of this perturbation. Moreover, PI3K-independent pathways may play significant roles in the dysregulation of insulin signaling and inflammatory effects.

This study was performed in order to determine whether or not hyperinsulinemia increases the effects of TNFα-stimulated expression of VCAM-1 above that seen for TNFα alone and which molecular pathways in particular mediate this effect. We report here that insulin- and TNFα-stimulated VCAM-1 expression appears to be regulated by the c-jun N-terminal kinase pathway as demonstrated by decreased VCAM-1 expression. Additionally, hyperinsulinemia augments TNFα-stimulated VCAM-1 expression above that seen for TNFα alone. Third, inhibition of the PI3K pathway, a hallmark of insulin signaling dysregulation, significantly increased insulin plus TNFα induced VCAM-1 expression; thus, implicating the pleiotropic effects of the PI3K pathway. Finally, we not only show that insulin or TNFα alone stimulate nuclear import of NFκB, but also show that in the presence of insulin and TNFα together, there are greater amounts of NFκB translocated to the nucleus and sooner than seen with insulin- or TNFα-stimulated NFκB alone.

## Methods

### 2.1. Materials

All general lab reagents were purchased from Sigma-Aldrich (St. Louis, MO.). Primary antibodies to VCAM-1 proteins were from Cell Signaling Technology (Boston, MA) and BD Biosciences (San Jose, CA). Primary antibodies to NFκB p65 (Cat# 4764) were from Cell Signaling (Boston, MA). PVDF membranes and other Western blot accessories were from GE Healthcare/Amersham (Piscataway, NJ) and the secondary HRP-conjugated and FITC-conjugated antibodies were from Santa Cruz Biotechnology, Inc. (Santa Cruz, CA). Vascular endothelial cells (VEC) were rat aorta vascular cells purchased from ATCC (Manassas, VA) (Cat# CRL 1446) and maintained in culture medium from Gibco/Invitrogen (Carlsbad, CA). Kinase inhibitors PD98059 (for MEK1/2) and Wortmannin (for PI3K) were from Cell Signaling (Danvers, MA). SB203580 (p38 MAPK inhibitor) and SP600125 (JNK inhibitor) were from EMD/Calbiochem (Gibbstown, NJ). TNFα was from Roche Applied Science (Indianapolis, IN) and insulin was from Sigma-Aldrich (St. Louis, MO). NE-PER nuclear extraction kit (Cat# P78835) was from Thermo-Fisher (Pittsburg, PA).

### 2.2. Cell Culture

VEC were cultured in growth medium [DMEM with 4 mM L-glutamine modified by ATCC to contain 4.5 g/L-glucose, 1.5 g/L sodium bicarbonate and supplemented with 10% heat-inactivated fetal bovine serum (Gibco/Invitrogen, Carlsbad, CA) and 1% Antimycotic-Antibiotic solution (Gibco)] and cultured at 37°C, 5% CO_2 _atmosphere from passages 1 - 10. VEC were then cultured in serum-free medium (SFM) for 24 h, pre-treated in the absence or presence of indicated inhibitors for an additional hour, and then incubated in SFM without or with insulin (10 nM) or TNFα (20 ng/mL) alone or in combination for designated times.

### 2.3 SDS-PAGE and Western Blot Analysis and Protein Expression

VEC were cultured in SFM for 24 h before any treatments were performed. Thereafter, cell monolayers were treated with or without designated inhibitors for one hour and then treated with insulin (10 nM), TNFα (20 ng/mL) or a combination of both for indicated times. Whole cell lysates were prepared using lysis buffer (50 mM HEPES, 150 mM NaCl, 15 mM MgCl_2_, 1 mM PIPES, 1 mM NaHPO_4_, 1 mM DTT, 1 mM Na Vanadate, 1% TX-100, 0.05% SDS, 10 μg/mol Aprotinin, and 10 μg/mol Leupeptin). Lysates were cleared and protein concentrations were determined in order to load lanes with equal amounts of protein. Equal protein amounts were placed in 2 × Laemmli Sample Buffer, frozen overnight and then boiled for 5 minutes just before use. Forty microliters of cleared lysates plus sample buffer were loaded into each well of an 8-16% Pierce Precise Protein gel (Thermo-Fisher, Waltham, MA) and were run in 1 × Tris/HEPES/SDS running buffer at 100 V for 45 min. Proteins were then transferred to PVDF or nitrocellulose membranes (Millipore, Billerica, MA), using a standard wet transfer protocol. After completion of protein transfer, membranes were washed two times in 1 × tris-buffered saline-tween (TBS-T) solution for 10 min. Membranes were then incubated in 5% bovine serum albumin (BSA) in 1 × TBS-T blocking solution for 2 h at room temperature, washed 2 times in 1 × TBS-T for 5 min and then incubated with a designated primary antibody solution (1:1000 in 1% BSA/TBS-T) overnight at 4°C. Membranes were washed 3 times with TBS-T and then incubated with a designated secondary antibody (1:2000 in 1% BSA/TBS-T) conjugated with horseradish peroxidase at room temperature for 2 hours. Membranes were washed 2 times with TBS-T for 10 min at room temperature and washed once with 1 × TBS for 10 min. One milliliter of ECL (GE/Amersham) detection solution was added to each membrane and incubated for 1 min. Excess ECL was removed and membranes were exposed to HyperFilm (GE/Amersham) for visualization of proteins. Densitometry analysis was performed using the ImageQuant TL v2005 (GE/Amersham) software program in order to quantitate profile bands on representative films.

### 2.4 Immunofluorescence

Immunfluorescence (IF) was performed to visualize the expression of VCAM-1 in VEC. VEC were cultured in 6-well plates containing BD Coat Coverslips (BD Biosciences, San Jose, CA) in complete growth medium. Subsequently, VEC were pre-incubated in serum-free medium (SFM) for 24 hours then treated without or with designated inhibitors and cytokines. After treatments, cells were rinsed twice with PBS then fixed with 2% paraformaldehyde for 15 min at room temperature. After fixation, cells were washed three times with PBS for 10 min at room temperature, washed once with 70% ethanol/PBS, once with 95% ethanol, and once with 100% ethanol each for 5 minutes at room temperature. Cells were then blocked with 10% Normal Donkey Serum solution for 2 h. VCAM-1 expression was detected using rabbit anti-VCAM-1 antibodies (Santa Cruz, CA) primary antibodies (1:50) and FITC-conjugated donkey anti-rabbit IgG (Santa Cruz Biotechnology, Santa Cruz, CA) secondary antibodies (1:100). Secondary antibodies alone were used to test for non-specific binding. After antibody staining, coverslips were then mounted on glass slides with Prolong Gold Anti-Fade Reagent (Invitrogen, Carlsbad, CA) and allowed to cure overnight at room temperature prior to visualization using the Zeiss Axioplan Digital Deconvolution Microscope (Carl Zeiss, Inc., North America) and Slidebook software program (Olympus, Center Valley, PA).

### 2.5 Inhibitors

Time course phosphorylation assays were first conducted on VEC in the presence of insulin or TNFα alone in order to determine the time points at which phosphorylation of intracellular kinase intermediates were activated. Subsequently, dose response analyses were performed in order to determine half maximal inhibitory concentrations (IC_50_) for inhibitors.

### 2.6 Flow Cytometry

Cells were grown in CGM until 75% confluence. Growth medium was replaced with serum-free medium (SFM) for 24 hours. Cells were incubated in SFM without or with insulin or TNF-alpha alone or in combination for indicated times. Cells were carefully lifted from culture plates using Nozyme (Sigma, St. Loius, MO.). Cell counts were performed using Trypan blue and a hemocytometer. Equal numbers of cells (2 × 10^6 ^cells) from each treatment group were placed into 1.5 mL Eppendorf tubes. Cells were centrifuged at 500 × g for 6 min. The supernatants were removed and cells were resuspended in 100 uL of SFM. Four microliters of VCAM-1/FITC conjugated primary antibodies (BD Biosciences, San Jose, CA) were added to each 100 uL of cell suspension containing 2 × 10^6 ^cells (otherwise 2 μL of antibody per 1 × 10^6 ^cells) and incubated on ice for 30 min. Two milliliters of Fluorescence Activated Cell Sorting (FACS) solution [1% BSA in PBS] were added to the cold cell antibody solution followed by centrifugation at 500 × g for 6 min. The supernatant was removed and cells were resuspended in two milliliters of FACS solution in order to remove non-specific binding antibodies. This process was repeated once more and supernatant was removed and replace with 200 μL of 1% paraformaldehyde (PFA) in FACS. The cell suspension was incubated on ice for 15 min and then read at 488 nm on a BD LSRII flow cytometer (BD Bioscience, San Jose, CA).

### 2.7 Nuclear and Cytoplasmic Fraction Separation Assays

We performed nuclear extraction methodology as per protocol instructions in the kit from Pierce/Thermo-Fisher (Cat# P78835). Briefly, once cells were treated with the designated inhibitors and/or insulin and TNFα, cells were washed with phosphate buffer solution (PBS) twice and then cells were dislodge in 1 mL of PBS and transferred to 1.5 mL centrifuge tubes. Cells were centrifuged at 4°C at 500 × g. Several lysis buffers were used to sequentially disrupt the plasma membrane only and then the nuclear membrane with intermittent centrifugations in order to isolate cytoplasmic and nuclear fractions of NFκB.

### 2.8 Statistical Analysis

Data were analyzed by either unpaired Student's *t *test (two groups) or ANOVA with subsequent Tukey post test (several groups) as indicated. A "P" value of less than 0.05 was considered significant. Results were expressed as the mean ± SEM of three or more independent experiments.

## Results

**Figure 1:**
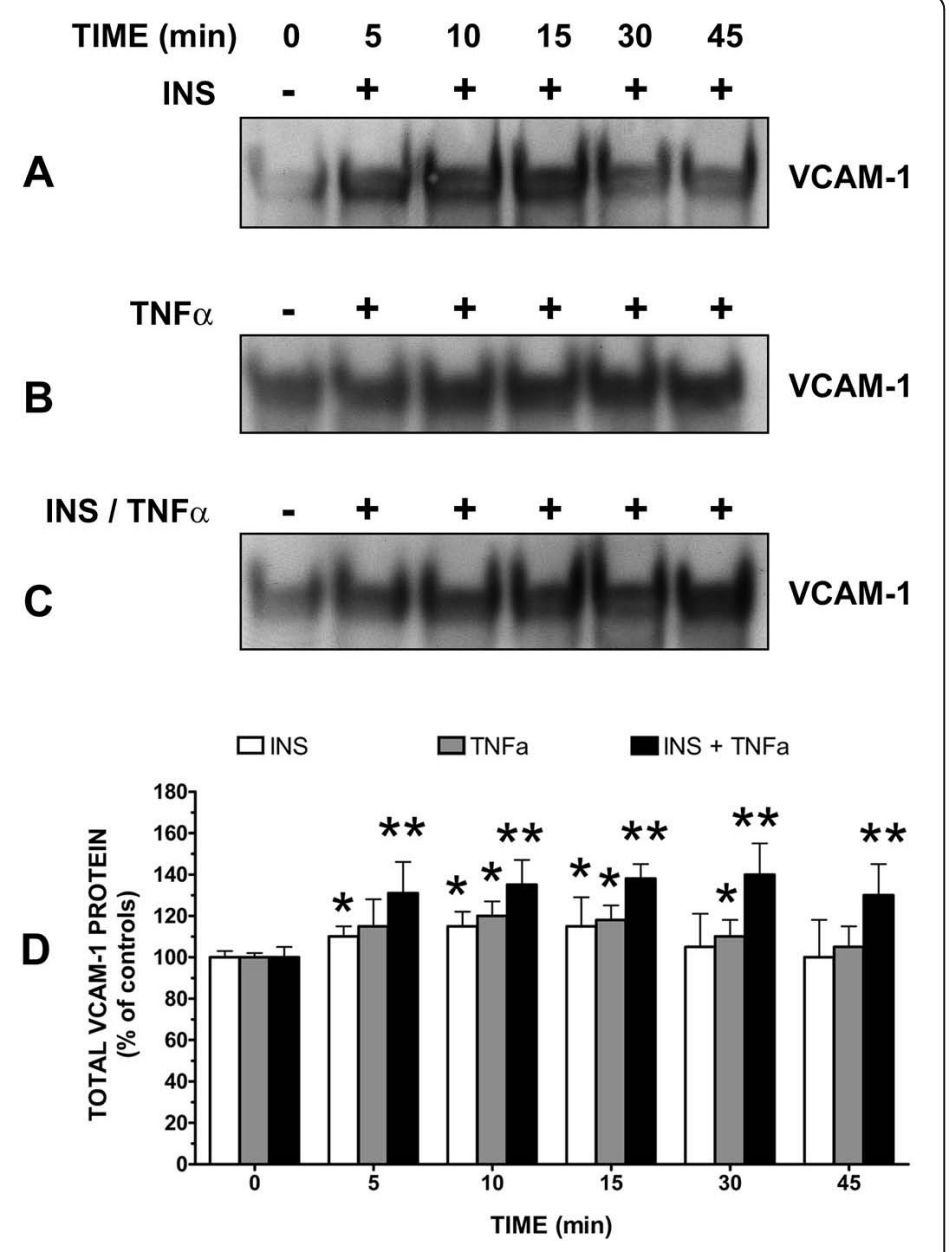
**Insulin and Tumor Necrosis Factor-alpha (TNFα) stimulate increases in VCAM-1 expression in vascular endothelial cells (VEC)**. Cells were incubated in growth medium until 80% confluent. Growth medium was removed from cultured cells and replaced with serum-free medium for 24 h. Cells were treated without or with [A] insulin (INS) (10 nM), [B] TNFα (20 ng/mL) or [C] both for designated times and VCAM-1 protein expression was determined via Western blot analysis. Western blots are representative profiles of six assays. [D] Changes in VCAM-1 protein are expressed as the percent of control and represent the mean ± standard error of the mean (SEM) for six independent experiments. *, P < 0.05 vs controls (serum-free medium alone); **, P < 0.05 vs TNFα alone.

Insulin (10 nM) (Figure 1A) or TNFα (20 ng/mL) (Figure 1B) alone moderately, but significantly (P < 0.05) increased VCAM-1 expression in vascular endothelial cells (VEC) in a time-dependent manner. More importantly in the presence of insulin and TNFα together, VCAM-1 protein content was significantly (P < 0.05) greater than that seen for insulin or TNFα alone (Figure 1C).

Since insulin- or TNFα-stimulated increases of VCAM-1 proteins were noted at short time periods we wanted to examine what time frames were necessary in order for insulin and TNFα to cause translocation of VCAM-1 protein from the peri-nuclear region to the cell surface, using immunofluorescence microscopy.

**Figure 2:**
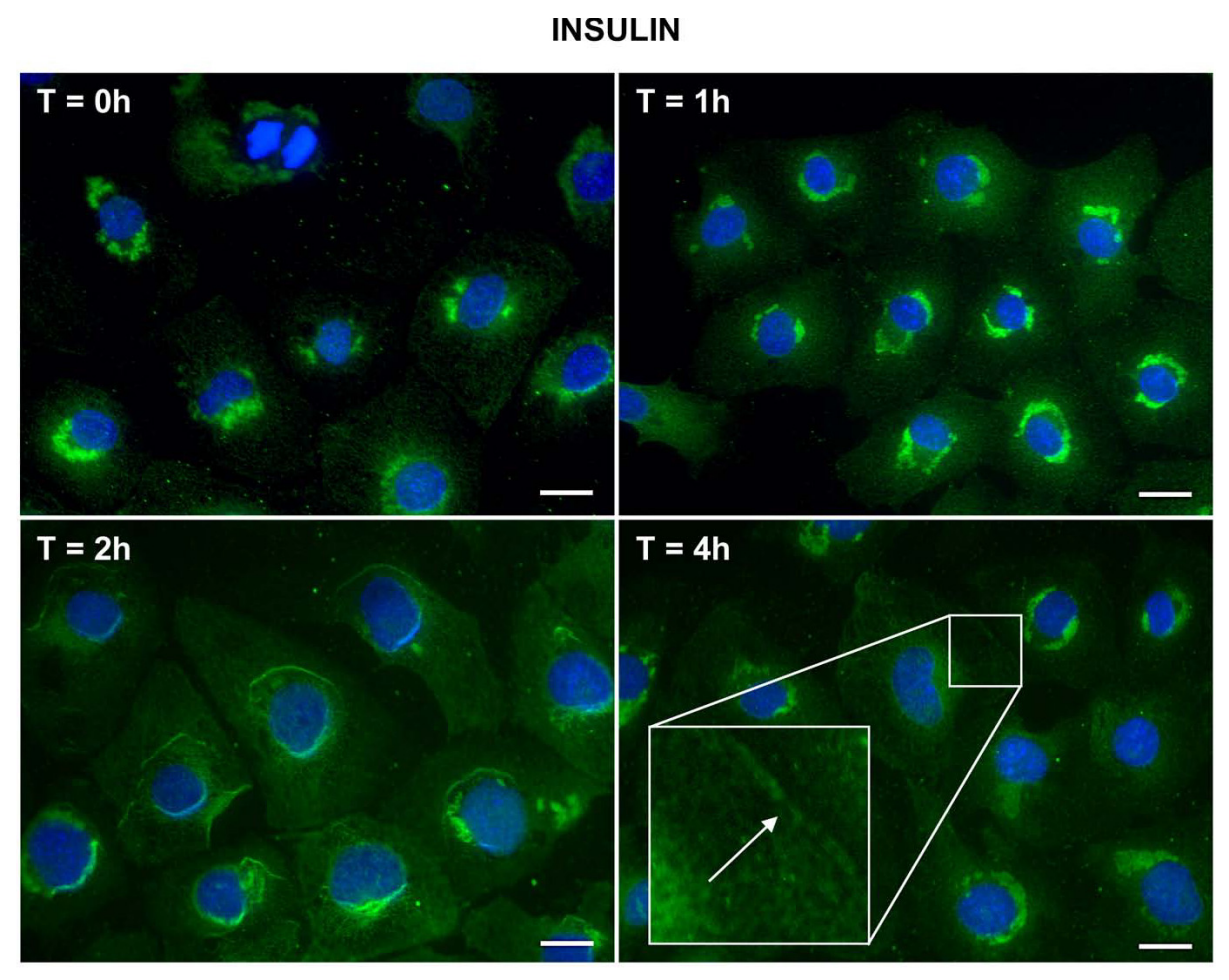
**Insulin stimulates VCAM-1 protein translocation from peri-nuclear to the cell surface in VEC**. Cells were plated onto round glass cover slips and allowed to proliferate in growth medium until 50% confluence. Growth medium was replaced with serum-free medium for 24 h. Cells were treated without or with insulin (10 nM) for indicated times. Cells were fixed and treated with primary and secondary antibodies as noted in the Methods section. Immunofluorescence was observed using deconvolution microscopy. VCAM-1 proteins are noted in green. Bars in pictures represent 20 μm.

**Figure 3:**
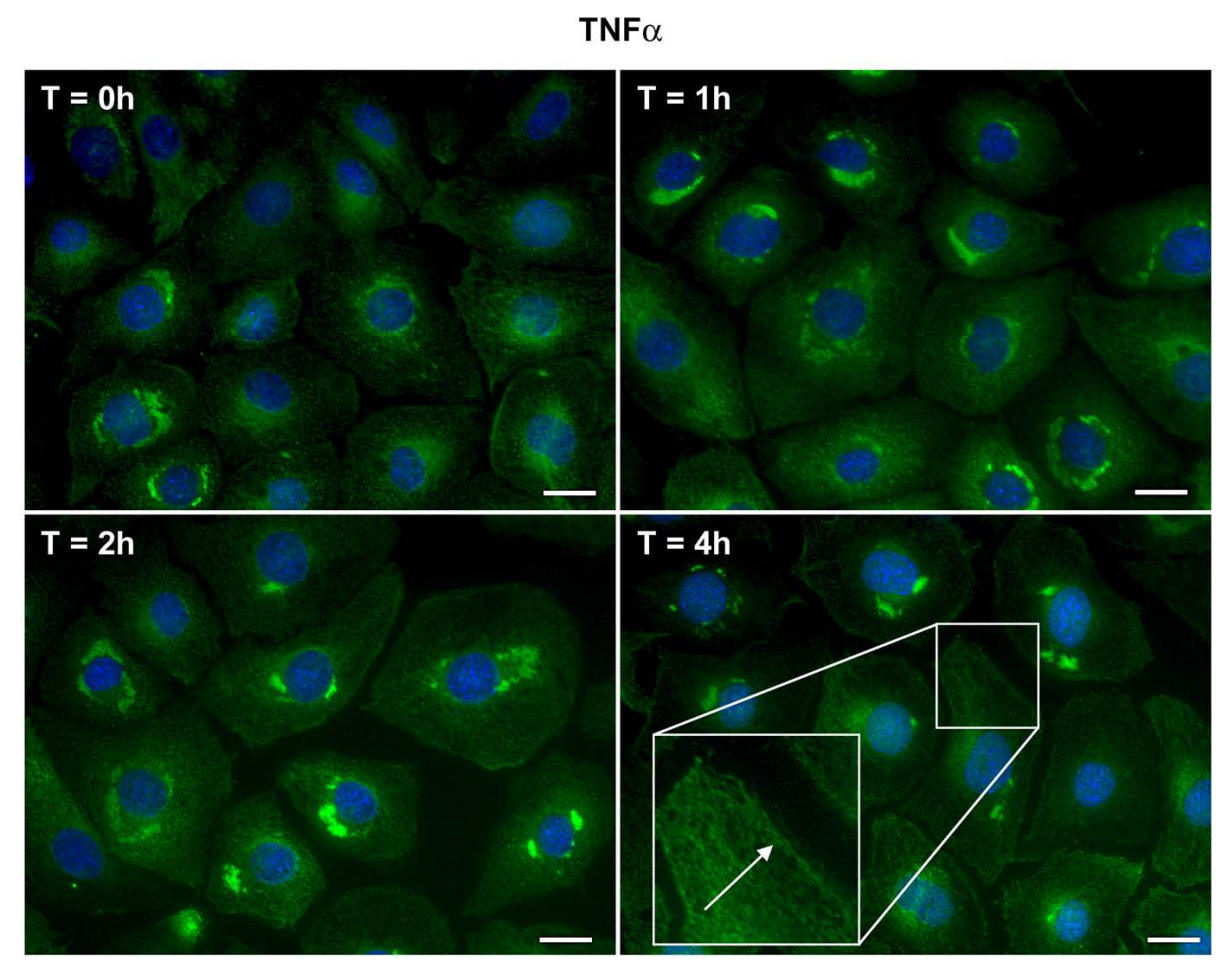
**TNFα stimulates VCAM-1 protein translocation from peri-nuclear to the cell surface in VEC**. Cells were plated onto round glass cover slips and allowed to proliferate in growth medium until 50% confluence. Growth medium was replaced with serum-free medium for 24 h. Cells were treated without or with TNFα (20 ng/mL) for indicated times. Cells were fixed and treated with primary and secondary antibodies as noted in the Methods section. Immunofluorescence was observed using deconvolution microscopy. VCAM-1 proteins are noted in green. Bars in pictures represent 20 μm.

**Figure 4:**
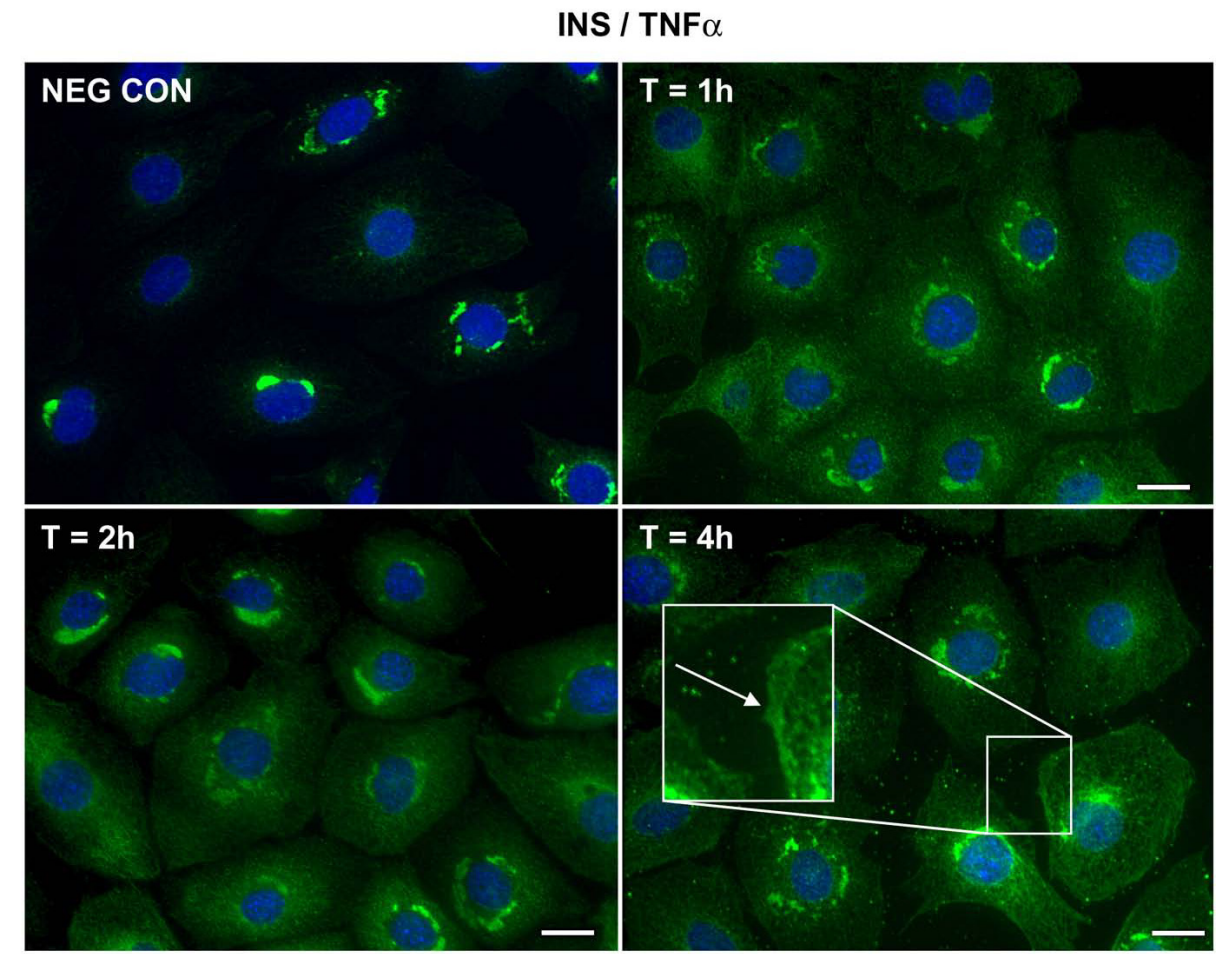
**Insulin plus TNFα stimulate VCAM-1 protein translocation from peri-nuclear to the cell surface in VEC**. Cells were plated onto round glass cover slips and allowed to proliferate in growth medium until 50% confluence. Growth medium was replaced with serum-free medium for 24 h. Cells were treated with insulin (10 nM) and TNFα (20 ng/mL) in combination for indicated times. Cells were fixed and treated with primary and secondary antibodies as noted in the Methods section. Immunofluorescence was observed using deconvolution microscopy. VCAM-1 proteins are noted in green. Negative control of secondary antibody alone is noted in the upper left quadrant. Bars in pictures represent 20 μm.

**Figure 5:**
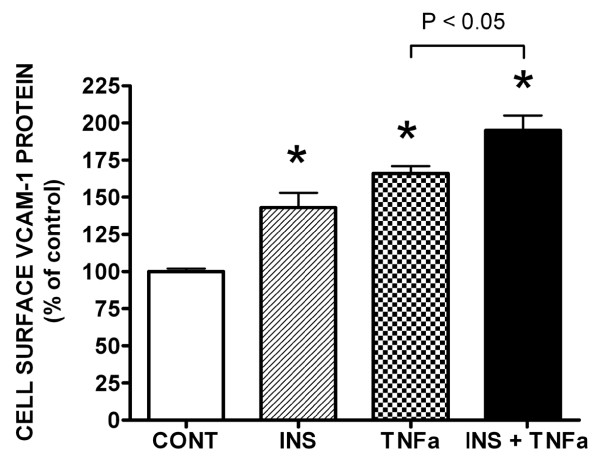
**Either insulin or TNFα alone or in combination stimulate VCAM-1 protein translocation from peri-nuclear to the cell surface in VEC**. Graph of flow cytometry results. Cells were prepared for flow cytometry as described in Methods. Increased cell surface VCAM-1 is expressed as percent of controls and represents the mean ± S.E.M. of four experiments. *, P < 0.05 vs controls (serum-free medium alone).

Insulin or TNFα alone stimulated the translocation of VCAM-1 from the peri-nuclear region to the cell surface. Unlike the short time frames that were observed associated with insulin and TNFα-stimulated increases in protein content, insulin (Figure 2) or TNFα induced (Figure 3) movement of VCAM-1 to the cell surface occurred over longer time periods; notably hours. Upon further observation, we noticed two interesting results. First, in the presence of insulin and TNFα together (Figure 4), the movement of VCAM-1 from the peri-nuclear region to the cell surface occurred in a shorter amount of time (one hour instead of two) as compare to that seen for insulin or TNFα alone. Second, we observed a stronger signal (i.e., an increased amount of VCAM-1) at the cell surface in the presence of insulin and TNFα together as compared to insulin or TNFα alone. Similar assays were performed using flow cytometry and similar results were observed (Figure 5).

Since insulin and TNFα are different biological molecules, we wanted to determine which intra-cellular pathways mediated insulin and TNFα-stimulated VCAM expression. In order to do so, we used PD98059 (MEK1/2 inhibitor), Wortmannin (PI3K inhibitor), SB203580 (p38 MAPK inhibitor) and SP600125 (c-jun N-terminal Kinase [JNK] inhibitor) to determine which kinase pathways were being activated by insulin and TNFα with reference to VCAM-1 expression.

**Figure 6:**
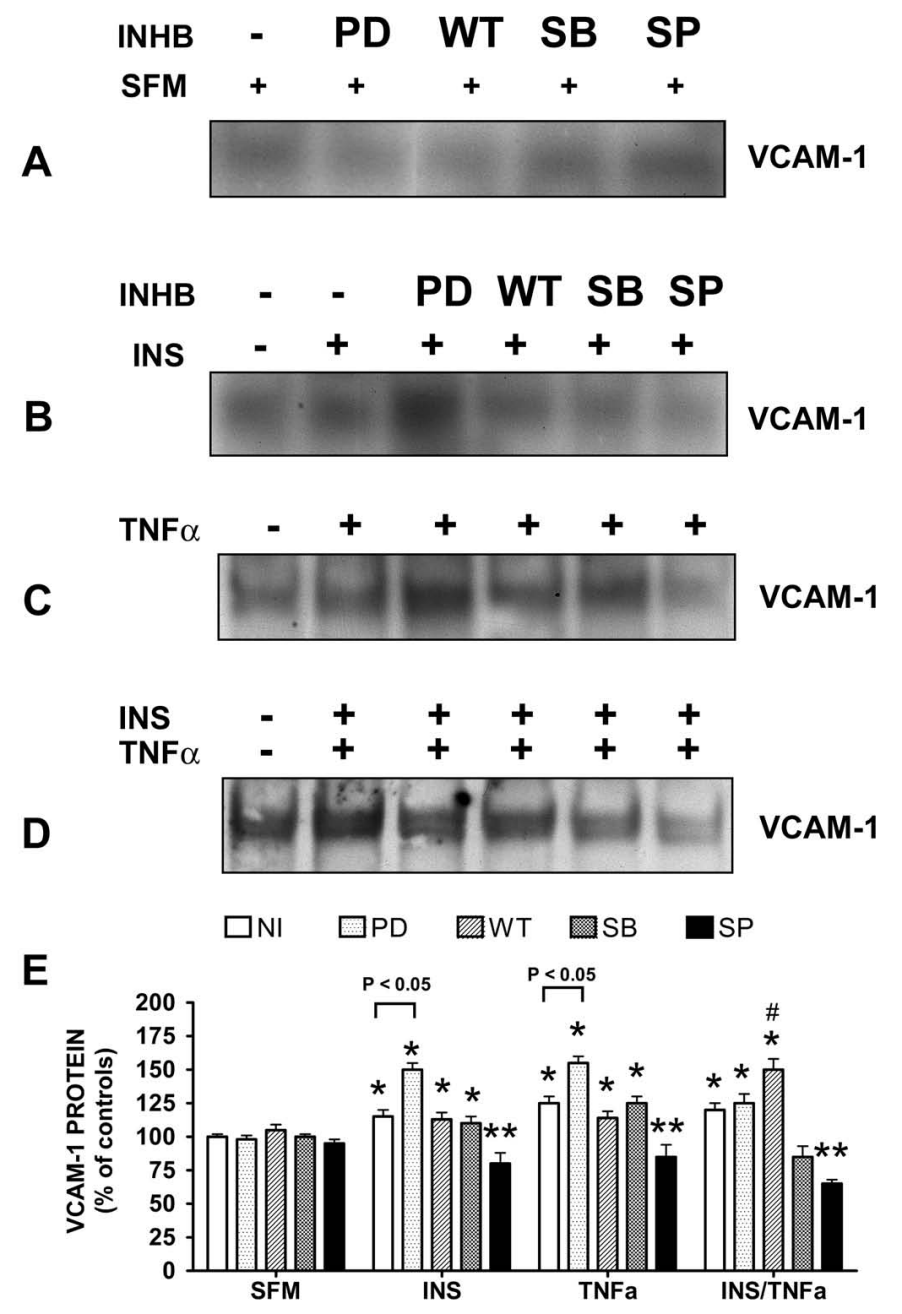
**The role of kinase pathways in VCAM-1 expression in VEC treated without or with insulin or TNFα alone or both and in the absence or presence of kinase inhibitors**. Cells were grown to 80% confluence in growth medium. Growth medium was replaced with serum-free medium for 24 h. Cells were pre-treated with either no inhibitor, PD98059 (10 μM), Wortmannin (15 μM), SB203580 (100 nM) or SP600125 (25 μM) for one hour and then treated without or with insulin (10 nM) or TNFα (20 ng/mL) alone, or insulin plus TNFα for 10 additional minutes. Proteins from cleared lysates were analyzed by SDS-PAGE and determined by Western blot analysis for VCAM-1 expression. Representative Western blots of VCAM-1 expression are shown in the absence or presence of indicated inhibitors [Panel A] and without or with insulin (INS) [Panel B] or TNFα [Panel C] alone, or without or with insulin plus TNFα [Panel D]. [Panel E, graph] Changes in amounts of VCAM-1 protein in the absence or presence of inhibitors and without or with insulin or TNFα, or insulin plus TNFα are expressed as percent of controls and are represented as means ± SEM of 5 experiments. *, P < 0.05 vs controls (serum-free medium alone); **, P < 0.05 vs positive controls (insulin or TNFα with no inhibitor). #, P < 0.05 vs insulin plus TNFα (no inhibitors). (SFM) serum-free medium; (INHB) inhibitors; (NI) no inhibitors; (INS) insulin; (TNFα) Tumor Necrosis Factor-alpha; (PD) PD98059; (WT) Wortmannin; (SB) SB203580; and (SP) SP600125.

Using the information gathered from our time-course and dose-response experiments (Table 1) we performed assays to determine the effects of these inhibitors on insulin or TNFα alone induced VCAM-1 expression or in the presence of both analogs (insulin plus TNFα) (Figure 6). We first examined the effects of the inhibitors alone. The kinase inhibitors had no effect on VCAM-1 expression after one hour of incubation time plus the additional ten minute "no analog/analog" treatment time (Figure 6A). However, in the presence of insulin or TNFα alone or in combination thereof, only the JNK inhibitor SP600125 significantly (P < 0.05) inhibited expression of VCAM-1 as compared to positive controls (i.e., designated analog with no inhibitor). Both the PI3K inhibitor Wortmannin and the p38 inhibitor SB203580 moderately inhibited insulin or TNFα-stimulated VCAM-1 expression, but without statistical significance.

**Table 1:** Kinase inhibitors designated by common nomenclature, noting target kinase and IC_50_

INHIBITOR	TARGET	IC 50 (μM)
PD98059	MEK1/2	10

WORTMANNIN	PI3K	15

SB203580	p38	0.1

SP600125	JNK	25

What were more interesting results were two events that seemed counter intuitive. The first event was the influence of PD98059 on VCAM-1 protein expression. The MEK1/2 inhibitor PD98059 significantly (P < 0.05) increased VCAM-1 expression 50% above controls (serum-free medium alone) in the presence of insulin (Figure 6B) or TNFα (Figure 6C) alone. However, in the presence of both insulin and TNFα (Figure 6D), the MEK1/2 inhibitor (PD98059) significantly (P < 0.05) increased VCAM-1 expression only 35% above controls (serum-free medium alone).

The second event that piqued our interest was the effect of Wortmannin on VCAM-1 expression in the presence of insulin and TNFα, together. In the presence of Wortmannin cells treated with insulin or TNFα alone showed no significant decrease in VCAM-1 expression as compared to controls (insulin or TNFα alone). However, in the presence of insulin and TNFα together, Wortmannin significantly (P < 0.05) increased VCAM-1 expression 50% above negative controls and 30% above positive controls (Figure 6D).

To further our investigation, we decided to examine the role of NFκB in insulin and TNFα-stimulated VCAM-1 expression. Since NFκB is correlated with inflammation, we wondered whether or not NFκB was regulated by insulin and/or TNFα and one of our selected kinase pathways. It has been shown that Hepatocyte Growth Factor (HGF) suppresses vascular endothelial growth factor-induced expression of endothelial VCAM-1 by inhibiting the NFκB pathway [15]. Thus, we wanted to determine the affects of insulin and TNFα on NFκB in VEC and whether or not our selected inhibitors would perturb this signaling.

**Figure 7:**
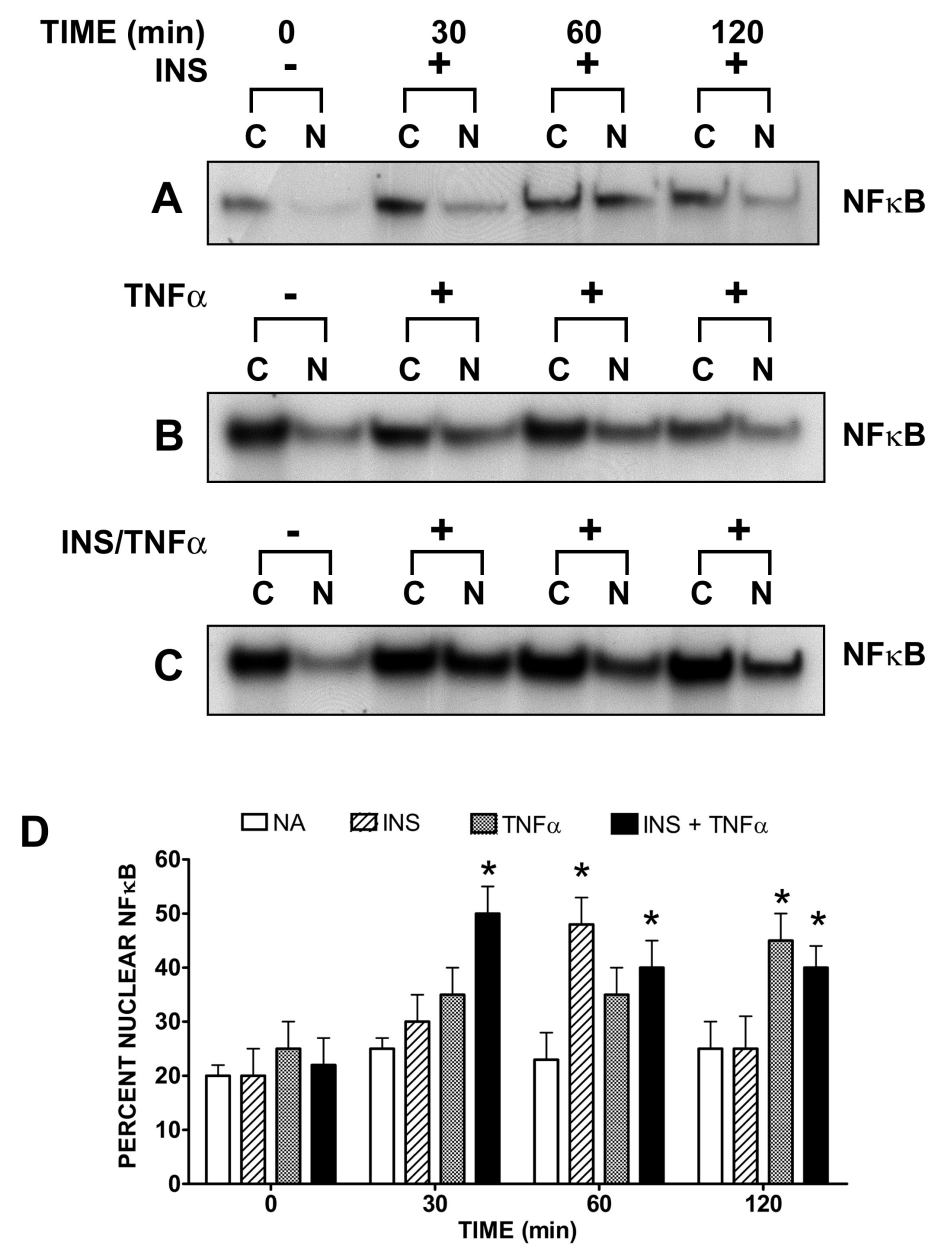
**Effects of insulin and/or TNFα on nuclear import of NFκB in VEC**. Cells were grown to 80% confluence in growth medium. Cultures were subsequently grown in serum-free medium for 24 hours and then treated without or with (INS) insulin (10 nM) or TNFα (20 ng/mL) alone or in combination for indicated times. Cytoplasmic and nuclear fractions were isolated and relative amounts of NFκB were determined. Representative western blots of cells treated without or with (INS) insulin [Panel A], TNFα [Panel B] or insulin plus TNFα (INS/TNFα) together [Panel C] are shown. Cytoplasmic (C) and nuclear (N) fractions are shown at indicated times. [Panel D] Relative amounts of NFκB are expressed as percent nuclear [(Nuc)/(Cyt + Nuc) * 100] at designated times and represent the mean ± SEM of 4 separate experiments. *, P < 0.05 vs controls (serum-free medium alone). (NA) no analog.

We observed that individually, insulin (Figure 7A) or TNFα (Figure 7B) significantly (P < 0.05) stimulated NFκB nuclear import by 60 and 120 minutes, respectively. Interestingly, in the presence of insulin and TNFα concurrently, nuclear import of NFκB was significantly (P < 0.5) increased at 30 minutes as compared to insulin or TNFα alone, and was sustained for a longer period of time (Figure 7C). Cells not treated with insulin or TNFα or both, exhibited no significant change over time as compared to negative controls (Figure 7D).

**Figure 8:**
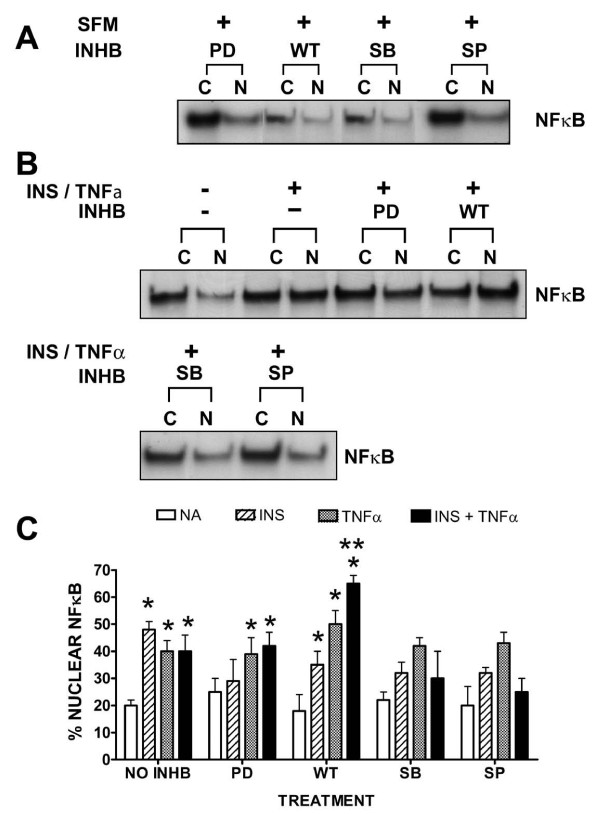
**Effects of kinase inhibitors on insulin augmented TNFα-stimulated NFκB nuclear import**. VEC were cultured in growth medium until 80% confluent and then cultured in serum-free medium for 24 h. Thereafter, cells were pre-treated with no inhibitor, PD98059 (10 μM), Wortmannin (15 μM), SB203580 (100 nM) or SP600125 (25 μM) for one hour and then treated with no analog (NA)(i.e., no insulin or TNFα) or with insulin (10 nM), TNFα (20 ng/mL) alone or in combination for 60 minutes. Representative Western blots at time 60 minutes are shown noting changes in relative NFκB protein content in cytoplasmic (C) and nuclear (N) fractions of cells treated with designated inhibitors alone [Panel A] or without or with inhibitors and in the absence or presence of insulin plus TNFα [Panel B]. [Panel C] Percentage of nuclear NFκB is expressed for cells treated with either no analog (NA) (open bars) or presence of insulin (striped bars) or TNFα (shaded bars) alone, or insulin plus TNFα (solid bars) and in the absence or presence of indicated inhibitor. Graph represents the mean ± SEM for 5 separate experiments. (NA) no analog; (NO INHB) no inhibitor; (INS) insulin; (TNFα) Tumor necrosis factor-alpha; (INS + TNFα) insulin plus TNFα; (PD) PD98059; (WT) Wortmannin; (SB) SB203580; (SP) SP600125. *, P < 0.05 vs controls (no analog no inhibitor; serum-free medium alone); **, P < 0.05 vs TNFα plus WT.

We then pre-incubated the cells with either no inhibitor, PD98059, Wortmannin, SB203580 or SP600125 for one hour and then treated the same cells with either no analog or with insulin and/or TNFα for 60 min. We first noted that the in the presence of the inhibitors alone (Figure 8A) there was no significant change in percent nuclear NFκB as compared to negative controls (Figure 8B, lane1). We also observed that in the presence of the inhibitors insulin or TNFα-stimulated NFκB nuclear import was either not affected or was moderately, but not statistically attenuated as compared to positive controls (not shown). In contrast, the PI3K inhibitor, Wortmannin, increased insulin plus TNFα-stimulated NFκB nuclear import significantly (P < 0.02) above that seen for positive controls (insulin or TNFα alone with no inhibitors) and significantly (P < 0.05) greater than that seen for TNFα alone with WT (Figure 8C).

## Disucussion

In the United States alone there are 23.6 million people reported to have diabetes with countless more undiagnosed individuals. T2DM is a syndrome of multiple disorders that starts with insulin resistance and eventually leads to a plethora of pathologies. Vascular complications are the most prevalent pathology found associated with T2DM. Insulin resistance and hyperinsulinemia are risk factors for cardiovascular diseases (CVD) [1,16], and in time and left unchecked CVD will lead to loss of limb and life. A particular vascular event that occurs in diabetes is increased expression of cellular adhesion molecules on the surface of vascular endothelial cells. VCAM-1 is one such adhesion molecule that is upregulated in the state of insulin resistance and hyperinsulinemia. Its upregulation leads to inflammatory effects and remodeling of the vessel, contributing to the occlusion of the arterial lumen.

VCAM-1 plays an important role in immune responses. Some have reported that this cell surface molecule is important in bacterial and viral defense mechanisms [17]. Yet, more importantly is its role in inflammation. VCAM-1 binds to integrins, which are found on the surface of cells belonging to the immune system such as leukocytes, lymphocytes and monocytes [18]. These immune-response cells are the scavengers of the circulatory system. When they are activated, they express their own surface adhesion markers, which in turn are recognized by cellular adhesion molecules that are located on the surface of the endothelial cells of the intima. Activated immune cells translocate into the vascular tissue and away from the circulatory system whereby they secrete inflammatory cytokines and induce the sequelae of inflammation and vascular wall remodeling.

Another aspect of VCAM-1 activity has been observed in the presence of oxidized lipids [10]. Oxidized lipids assist in the upregulation of VCAM-1 expression, whereby adhesion of leukocyte and monocytes to the endothelium membrane increases [18]. In both cases, transendothelial migration of leukocytes and monocytes occurs, causing the secretion of inflammatory cytokines and chemokines. One such cytokine is TNFα, which not only is secreted by mature monocytes (i.e., macrophages), but also by perturbed endothelial cells [19].

TNFα is a well characterized inflammatory cytokine and its presence is strongly correlated with atherogenesis [8,20]. In contrast, insulin's role in atherogenesis has been a hotly debated topic. On the one hand, some contend that insulin stimulates the increase of nitric oxide (NO) and decreases systemic inflammation [5,21], which in turn may inhibit atherogenesis. On the other hand, others argue that insulin, especially in the context of hyperinsulinemia, increases the proliferation of endothelial and vascular smooth muscle cells and exacerbates the inflammatory response [22,23]. Yet, if one looks at this argument, the two sides are not contradictory. Insulin appears to have a "double-phase" effect. At physiologic concentrations, insulin has a vascular protective effect [5]. However, at hyper-physiologic concentrations, insulin appears to have a vascular insult effect, by augmenting the effects of deleterious cytokines. It has been demonstrated that high physiologic insulin levels may inhibit endothelial progenitor cell proliferation [24,25]. In contrast, hyper-physiologic insulin appears to augment more potent growth factors such as platelet-derived growth factor [12,26] thereby causing a destructive effect on the endothelium.

VCAM-1 plays a more dominant role in atherosclerosis than ICAM-1 [27]. Yet, both appear to increase in expression within the context of insulin resistance and hyperinsulinemia. One possible mechanism for increased expression of CAMs is insulin's ability to prime cells to be more responsive to more potent cytokines, which in turn complement the insulin resistant state and thereby increase CAM expression [28]. Previous studies have associated hyperinsulinemia with atherosclerosis [29-31]. Additionally, CAM expression has been linked to inflammatory conditions that appear to be correlated with atherosclerosis [32,33]. Other investigators have posited that insulin resistance and hyperinsulinemia may contribute to the increased expression of TNFα, whereby pro-inflammatory mechanisms are increased in the presence of insulin resistance [34].

We have shown in this study that high-physiological concentrations of insulin increase the expression of VCAM-1 above that seen for quiescent cells. In comparison, TNFα alone stimulates increases in the expression of VCAM-1 above that seen for controls and insulin. What is impressive is the affect of insulin on TNFα-stimulated increases in VCAM-1 expression. In the presence of insulin and TNFα concurrently, stimulation of VCAM-1 expression is greater than that seen for TNFα alone. Additionally, in the presence of insulin and TNFα simultaneously, translocation of VCAM-1 protein from peri-nuclear regions to the cell surface occurs faster than that seen with cells in the presence of either insulin or TNFα alone.

The path from hormone and cytokine to intracellular kinase pathway has always been intriguing and there is no exception in this study. In what appears to be simple inhibitor assays, the JNK inhibitor SP600125 appears to be the major regulated kinase pathway for insulin and TNFα stimulation of VCAM-1 expression. Yet, what became most interesting in this study were the changes in VCAM-1 expression in cells pre-treated with PD98059 (MEK1/2 inhibitor) and Wortmannin (PI3K inhibitor). In the presence of insulin or TNFα alone, PD98059 stimulated an increase in VCAM-1 expression 50% above controls. In the presence of insulin or TNFα alone, Wortmannin had moderate, but statistically insignificant, inhibition of insulin- or TNFα-stimulated VCAM-1 expression. In contrast, in the presence of insulin and TNFα together, cells pre-treated with Wortmannin exhibited a 50% increase in VCAM-1 expression above negative controls (serum-free medium alone) and 30% above that seen for insulin and TNFα together in the absence of an inhibitor. Taken together, these results indicate that (1) cross talk occurs among the major kinase pathways, (2) inhibition of one kinase pathway may disinhibit another, and (3) hyperinsulinemia in conjunction with insulin resistance's canonical perturbed pathway (PI3K) exacerbates TNFα-stimulated increases in VCAM-1.

Since insulin and TNFα appear to be associated with inflammation of the arterial endothelium, we were interested in determining whether or not inhibition of any of the major intracellular kinase pathways that were stimulated by insulin and TNFα would affect the canonical inflammation player, NFκB. We used kinase inhibitors to determine their influences on insulin and TNFα stimulated nuclear import of NFκB in endothelial cells. While MEK1/2, p38 and JNK inhibitors reduced insulin and TNFα-stimulated NFκB nuclear import, the PI3K inhibitor Wortmannin significantly (P < 0.05) increased NFκB nuclear import in cells treated with insulin or TNFα. In fact, in the presence of both insulin and TNFα, nuclear import of NFκB was greater than that seen in the presence of insulin or TNFα alone and occurred more quickly than that seen for either one alone.

It should be kept in mind that these studies were performed in a rat aorta vascular endothelial cell line. Thus, it would be of interest to perform the same experiments in primary cell cultures in order to determine whether or not these observations hold true for primary endothelial cells as well. Thus, future studies are warranted.

Taken together, these results indicate that insulin can be atherogenic, can augment TNFα-stimulated VCAM-1 expression and thus play a key role in vascular inflammation. Additionally, our data show that although insulin- and TNFα-stimulated increases of VCAM-1 appear to be regulated by the JNK pathway, perturbation of the PI3K pathway (a common insulin resistant effect) greatly increased the amount and rate of insulin and TNFα-stimulated NFκB nuclear import; a precursor event to increased inflammatory sequelae.

## Conclusions

Hyperinsulinemia and a perturbed PI3K pathway appear to augment TNFα stimulation of VCAM-1 protein, VCAM-1 translocation to the cell surface and nuclear import of NFκB. These results taken together indicate that hyperinsulinemia, insulin resistance and perturbed PI3K signaling, which are hallmarks of Type-2 Diabetes Mellitus, may exacerbate the inflammatory conditions that are affected by TNFα and may play important roles in the pathogenesis of atherosclerosis.

## Abbreviations

CAM: Cellular Adhesion Molecule; CGM: Cell Growth Medium; ERK: Extracellular Signal-Regulated Kinase; FACS: Fluorescence Activated Cell Sorter; IC_50_: Half Maximal Inhibitory Concentration; JNK: c-jun N-terminal Kinase; MAPK: Mitogen-activated Protein Kinase; NFκB: Nuclear Factor kappa-B; PBS: Phosphate-buffered Saline; PI3K: Phosphatidylinositol-3 Kinase; PVDF: Polyvinylidene Fluoride; SFM: Serum-free Medium; T2DM: Type-2 Diabetes Mellitus; TNFα: Tumor Necrosis Factor-alpha; VCAM-1: Vascular Cell Adhesion Molecule-1; VEC: Vascular Endothelial Cells.

## Competing interests

The authors declare that they have no competing interests.

## Authors' contributions

DM carried out the Western blot, immunocytochemistry and flow cytometry experiments. Additionally, DM gathered the raw data and using statistical analyses and graphic programs compiled the data into meaningful information. DM also contributed to writing the Methods section of the manuscript. MG collected all of the graphics and formed the figures of the manuscript. MG contributed to writing the Methods section and wrote the remainder of the manuscript. All authors have read and approved this manuscript.
